# Comprehensive Assessment of Orofacial Health and Disease Related Parameters in Adolescents with Juvenile Idiopathic Arthritis—A Cross-Sectional Study

**DOI:** 10.3390/jcm9020513

**Published:** 2020-02-13

**Authors:** Cordula Leonie Merle, Robin Hoffmann, Jan Schmickler, Michael Rühlmann, Nadia Challakh, Rainer Haak, Gerhard Schmalz, Dirk Ziebolz

**Affiliations:** 1 Department of Cariology, Endodontology and Periodontology, University of Leipzig, 04103 Leipzig, Germany; cordulaleonie.merle@medizin.uni-leipzig.de (C.L.M.); schmickler.jan@gmail.com (J.S.); Nadia.Challakh@medizin.uni-leipzig.de (N.C.); rainer.haak@medizin.uni-leipzig.de (R.H.); gerhard.schmalz@medizin.uni-leipzig.de (G.S.); 2 Dental Practice Pröpper, Flachsenberg, Hoffmann, 34119 Kassel, Germany; Robin.Hoffmann1@gmx.de; 3 Medical Practice for Pediatricy And Pediatric Rheumatology M. Rühlmann, 37073 Goettingen, Germany; ruehlmann@med.uni-goettingen.de; 4 Department of Pediatricy II (Pediatric Neurology), University of Goettingen, 37075 Goettingen, Germany

**Keywords:** dental care, juvenile idiopathic arthritis, temporomandibular joint, oral health, oral and systemic disease interaction

## Abstract

Background: The aim of this cross-sectional study was to investigate oral health and functional status of adolescents with juvenile idiopathic arthritis (JIA) and its possible link to disease specific parameters. Methods: Patients with JIA were recruited (November 2012–October 2014) and disease specific information was extracted from patients’ records. Oral examination included: dental findings (decayed-, missing- and filled-teeth-index (dmf-t/DMF-T)), gingival inflammation (papilla-bleeding-index (PBI)) and periodontal screening index (PSI). Functional examination followed Research Diagnostic Criteria for Temporomandibular Disorders (RDC/TMD). Additionally, modified Helkimo’s Clinical Dysfunction Index and radiographic scoring were recorded. Results: 59 JIA patients were included. The mean dmf-t/DMF-T was 2.6. Only one patient showed no signs of gingival inflammation, while 57.6% had a maximum PSI of 2 or less. Positive functional findings were assessed clinically in more than half of the patients. Major diagnosis by RDC/TMD was osteoarthrosis. Patients with at least one positive anamnestic or clinical functional finding revealed significantly higher radiographic scores (CI = 0.440, *p* = 0.022). Patients with increased c-reactive-protein had a significantly higher PBI (Z = –2.118, *p* = 0.034) and increased radiographic scores (CI = 0.408, *p* = 0.043). Conclusions: Adolescents suffering from JIA show high levels of caries experience and gingival inflammation. Temporomandibular joint dysfunction is often seen in JIA patients. Consequently, special dental care programs would be recommendable.

## 1. Introduction

Juvenile idiopathic arthritis (JIA) is the chronic rheumatic arthritis with disease onset in childhood or youth. The definition includes idiopathic arthritis of one or more joints for more than six weeks (chronic) before the age of sixteen [1]. It is a collective term for a group of different diseases. Various subcategories are defined particularly by the number of affected joints (oligoarthritic or polyarthritic types), the presence of other symptoms and different blood parameters [1]. The prevalence is 0.03% among Caucasians. More girls (0.02%) than boys (0.01%) are affected [2].

JIA should be a disease of special dental interest: The temporomandibular joint (TMJ) is involved in 40% to 93.33% of patients suffering from JIA [3]. The inflammation can lead to growth disturbances and thereby to severe orthodontic findings as mandibular micro- and retrognathia, anterior open bites and asymmetries [4,5]. In the majority of cases the involvement of TMJ is painless and therefore lately diagnosed [6]. However, the TMJ is often already involved at disease onset [6]. Thus, dentists have a key role in the early diagnosis process. 

Furthermore, patients with diagnosed JIA are thought to be high risk patients for oral diseases. There are many possible mechanisms of correlation between JIA and oral diseases. First of all, pathobiological mechanisms of this rheumatic disease might influence periodontal conditions as already well described for rheumatoid arthritis in adults [7,8,9,10]. Moreover, these effects can be caused by the medication as well as emotional or psychosocial concerns of a chronic disease. Further influences could be challenges in oral hygiene due to an involvement of the upper limbs or the TMJ, sugar containing medication [11] and changed salivary parameters [12,13,14,15]. 

Previous studies about JIA patients showed inhomogeneous and controversial results regarding oral diseases [12,13,16,17,18,19,20,21,22,23,24]. Some studies showed increased dental caries experience [24], gingival inflammation [16,18,19] and dental biofilm accumulation [12,17,19] whereas other studies could not confirm these results [12,13,16,17,18,19]. Only a few studies have investigated signs of periodontitis in JIA patients. Some of these showed a significantly increased clinical attachment loss [20,21] and probing depth [20]; others did not [22,23]. The existing studies were performed within different health systems and observed only some aspects of orofacial health. The complexity of JIA and its diverse interaction with oral and functional health was not considered appropriately, yet. 

Consequently, the aim of this study was a comprehensive assessment of the orofacial health of children and adolescents with JIA. Different aspects of oral health (dental, gingival, periodontal) and functional status (anamnestic, clinical and radiographic) were examined. Additionally, disease parameters, including duration of disease, affected joints, blood parameters and medication, were assessed as possible influencing factors. It was hypothesized that JIA patients show insufficient oral health and functional findings.

## 2. Methods

This clinical, monocentric cross-sectional study was reviewed and approved by the local ethics committee of the medical faculty of the Georg-August-University Göttingen, Germany (application No. 3/3/13). All participants and their legal guardians were informed verbally and in writing about the study and provided their written informed consent for participation. Guidelines for ethical approvals for human subjects were followed in accordance with the Declaration of Helsinki. The recommendations for strengthening the reporting of observational studies in epidemiology (STROBE) [25] were considered.

### 2.1. Participants

Patients with diagnosed JIA treated by the Department of Pediatrics and Adolescent Medicine, University of Göttingen or by one practice for pediatrics and pediatric rheumatology Dr. M. Rühlmann in Göttingen received standardly a dental consultative examination in the Department of Preventive Dentistry, Periodontology and Cariology, University Medical Center Göttingen. Dental examination took place at the same day as the rheumatological assessment or not later than the day after. During the period of investigation (from November 2012 to September 2014) these patients were recruited for the study. Inclusion criteria were the diagnosis of JIA by a rheumatologist according to the criteria of the International League of Associations for Rheumatology (ILAR) [1] and an age between six and 18 years. The following exclusion criteria were defined: inability to undergo examination due to morbidity, immunosuppressive medication beside of JIA therapy, severe systemic disease in addition to JIA (e.g., diabetes mellitus, chronic heart diseases, endocarditis, delicate controlled hypertension, renal disorders, organ transplants, neuropathies, seizure disorders), infectious diseases (HIV/AIDS, hepatitis, tuberculosis), pregnancy, reduced fine motoric skills for other reasons than JIA, antibiotic therapy during the past three months. 

### 2.2. Recording of Subject Data

Participants’ health data were obtained from the patients’ records of the referring rheumatologist. The following aspects were registered: age, gender, medical history, time since diagnosis of JIA, number of affected joints in the first six months of disease (oligoarthritis: ≤4 joints, polyarthritis: >4 joints), any affected joints during whole disease duration, medication (current and past), current blood parameters if available (ANA: Antinuclear antibodies, HLA-B27: Human leukocyte antigen B27, RF: Rheumatoid factor, Anti-dsDNA: Anti-double stranded DNA antibodies, CRP: C-reactive protein). Cut-off titer of ANA was 1:80; RF was classified as positive if the current blood test was positive; CRP was rated as increased over 8 mg/L. 

Furthermore, the participants were asked to complete a standardized anamnestic questionnaire and a special functional anamnestic questionnaire. Questions were asked about functional limitations, joint sounds and parafunctions. 

### 2.3. Oral Examination

All examinations were conducted by one calibrated and experienced dentist. 

Dental findings (dmf-t/DMF-T): All teeth were assessed visually with mirror and probe. Due to the age of the participants, most were in transitional dentition. Accordingly, the combined dmf-t/DMF-T were calculated of all deciduous teeth and all permanent teeth, which were already more than half visible. All teeth showing or reasonably being suspected to have a cavitation reaching the dentine layer were assigned as d/D (= decayed). Teeth missing due to caries counted as m/M (= missing), filled and crowned teeth as f/F (= filled) [26]. 

Gingival inflammation (PBI): Gingival inflammation was assessed by papillary bleeding index (PBI). The examination was performed with a periodontal probe (PCP/UNC 15, Hu-Friedy, Chicago, IL, USA). It grades from 0 (no bleeding = gingiva without signs of inflammation) to 4 (profuse bleeding = severe gingival inflammation) [27]. 

Periodontal screening (PSI/PSR): The periodontal screening index/record was used to assess the periodontal situation. This score rates probing depth (<3.5 mm/3.5–5.5 mm/>5.5 mm), bleeding on probing (yes/no) and calculus (yes/no) per sextant. In children and adolescents only the teeth 16, 11, 26, 36, 31 and 46 were considered. The score was evaluated at 6 points per tooth using the WHO probe (Morita, Kyoto, Japan) [28,29]. 

Functional examination: Functional examination was performed corresponding to the Axis I (somatic findings) of the Research Diagnostic Criteria for Temporomandibular Disorders (RDC/TMD). Therefore, the patients were interviewed about pain and the following items were assessed: opening pattern, vertical range of motion and pain during opening, joint sounds, excursions and joint sound during excursions, muscle and joint pain with palpation [30]. The modified Helkimo’s Clinical Dysfunction Index was calculated afterwards [31,32]. 

Radiographic scoring: Temporomandibular joints were evaluated in available panoramic radiographs using a scoring system:

- Score 0: normal joint, convex shaped condyle;
- Score 1: slight deviation of the condyle from the convex shape;
- Score 2: Clearly flattened or deformed condyle;
- Score 3: Missing or shortened, flattened condyle.

Both right and left joints were assessed. One examiner scored each joint for three times. The most frequent score was included. Radiographs, in which not both joints could be evaluated clearly, or anomalies appearing beside the definitions of the score were excluded. 

### 2.4. Statistical Analysis

Statistical analyses were performed using the software SPSS Statistics (version 19, IBM). Descriptive statistics are reported as the means and standard deviation (SD) or median and range. The nonparametric tests Mann–Whitney *U* test or Kruskal and Wallis *H* test were used for comparison of continuous data and Chi²-test or Fisher’s exact test for categorical data. For analyzing possible correlations Cramér’s V based on Pearson’s chi-squared statistic, Kendall rank correlation coefficient or Pearson’s correlation coefficient have been used. The level of significance was defined as *p* < 0.05 for tests (two-sided).

## 3. Results

### 3.1. Patients

In total, 59 patients diagnosed with JIA (male = 17; mean age = 12.7 ± 3.1) were included. The mean duration of disease was 5.1 years. Table 1 shows the characteristics of the patients including disease specific information as medication, blood parameters and affected joints.

### 3.2. Oral Examination

Dental and periodontal findings: The mean dmf-t/DMF-T in all participants amounted to 2.6. On average, 0.9 teeth were decayed and 1.6 filled. Extended orthodontic appliances precluded measurement of gingival inflammation in four and of periodontal screening index in two cases. Only one patient showed no signs of gingival inflammation. A total of 57.6% had a maximum PSI of 2 or less (Table 1). 

Anamnestic, clinical and radiographic functional findings: Functional findings are shown in Table 2. Nearly 30% reported corresponding pain in the past. In total, positive findings were assessed clinically in more than half of the patients. Positive anamnestic functional findings correlated significantly with positive clinical functional findings (CI = 0.372, *p* = 0.007). Major diagnosis by RDC/TMD was Osteoarthrosis. Patients with at least one positive anamnestic or clinical functional finding revealed significantly higher radiographic scores (CI = 0.440, *p* = 0.022). All patients with radiographic score 2 or 3 showed anamnestic or clinical positive functional findings. 

**Figure 1:**
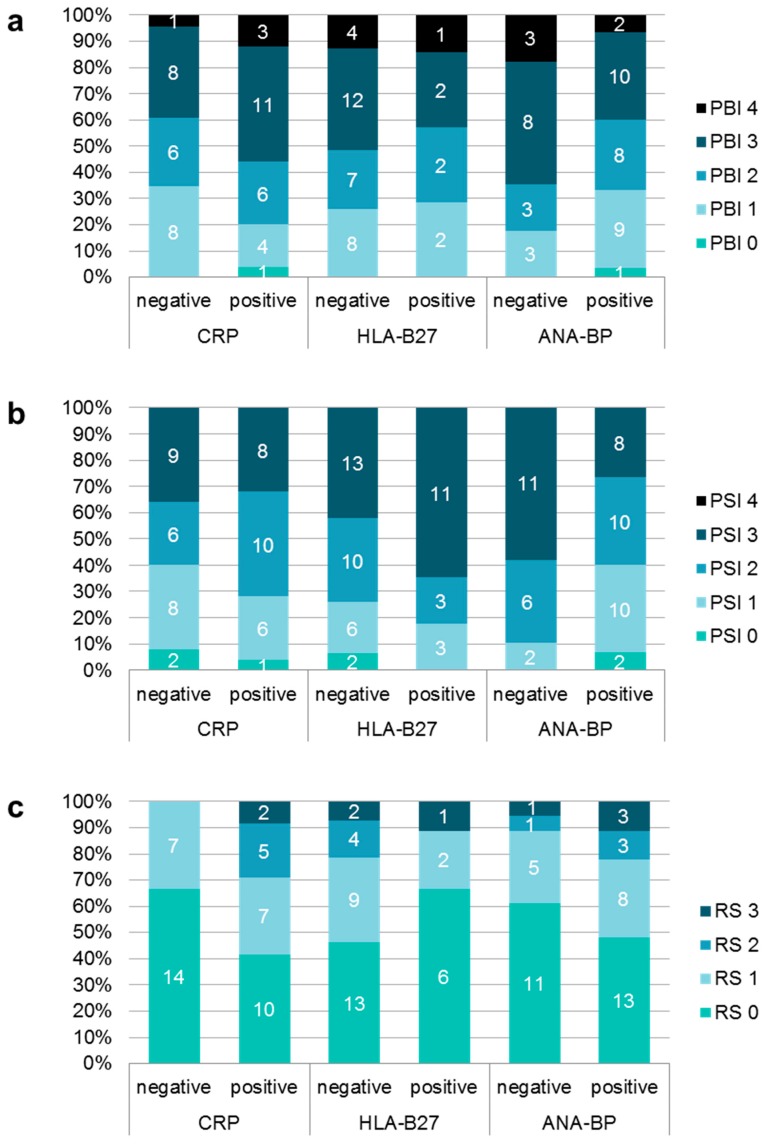
Distribution of different scores of papilla bleeding index (PBI); (**a**), periodontal screening index (PSI); (**b**) and radiographic score (RS); (**c**) depending on different blood values.

Association of disease parameters with oral findings: Some blood parameters showed associations to oral findings (Figure 1): Patients with increased CRP had a significantly higher PBI (Z = –2.118, *p* = 0.034). In contrast patients with increased ANA showed significantly less gingival inflammation (Z = –2.541, *p* = 0.011). Regarding the maximum PSI elevated scores were measured more often in patients with normal ANA and in HLA-B27-positive-patients. Radiographic scores were significantly increased in patients with positive CRP (CI = 0.408, *p* = 0.043). Neither affected joints nor limited mouth opening nor anamnestic and clinical pain showed a significant association to increased caries burden (Table 3). Duration of disease also showed no association to dmf-t/DMF-T.

## 4. Discussion

Summary of the main results: Only one participant showed no gingival inflammation. Signs of temporomandibular dysfunction were assessed in more than half of the patients. Some JIA-specific blood parameters were associated with worse oral and radiographic TMJ deformities. No association of other disease parameters, as affected joints or mouth opening capacity, to dental findings could be revealed.

Interpretation in comparison with the available literature: The current study did not investigate a healthy control group. Nevertheless, the results can be interpreted and discussed in relation to epidemiologic data from different German populations (Table 4). The fifth German oral health study [33] (DMS V) gives representative epidemiological data for the German general population, including dental caries and gingival inflammation, for 12-year-old adolescents. Obviously, JIA-patients in the current study had higher caries experience than the epidemiological data: On average, every JIA-patient had about two teeth more with caries experience and one tooth with untreated caries. Other studies reporting this effect of increased caries experience showed clearly higher values than the current study [24]. On the other hand, recent studies could not find differences in the dmf-t/DMF-T [12,13,16,17,18,19]. It is thereby conspicuous that studies with high caries experience [24] were performed before 2000, while caries prevalence in general has been reduced during past years [33]. Potential further reason could be assumed to be a decrease in sugar-containing medication. The higher amount of untreated caries corresponds to other studies [16,19]. This might be caused in the effect of less control-oriented dental visiting of patients with chronic diseases. 

The score for gingival inflammation showed an increase of one point in JIA-patients in this study compared to the general healthy population [33] (Table 4). Significantly higher presence of gingival inflammation [16,18,19] and dental plaque [12,17,19] have been reported but not by all available studies [12,13,16,18]. Reasons for the increased gingival and periodontal inflammation might be a generally increased inflammatory potential in rheumatic diseases [34] as well as affected oral behavior caused by general disease burden. Periodontitis in JIA patients has so far only been investigated in adolescent cohorts. The available data are inhomogeneous: Some studies showed significantly increased periodontal burden [20,21] others did not confirm these results [17,22,23]. The current study only investigated the periodontal screening index, making a statement about periodontal burden (bone loss) impossible. In any case, about forty percent had a PSI score 3 in at least one sextant. This is clearly higher than about 25% in another study [21]. Two studies measuring the probing depth showed significant differences between JIA patients and controls [17,20]. However, it is important to consider that increased probing depths in juvenile dentures can also be caused by the dentition and swollen gingiva. Therefore, one study showed hyperplasia in the JIA patients with a significant difference of the distance from the gingival margin to the cemento-enamel junction between JIA patients and controls [22]. A higher risk for periodontitis of the JIA-patients can be expected as this is well investigated for other rheumatic diseases. Patients with rheumatoid arthritis suffer from more severe attachment loss and increased tooth loss [10]. The possible causal mechanism [35] could concern JIA-patients in similar way. A probable reason why this association could not be revealed that clearly for JIA-patients is the low age of the investigated patients (11.9 to 15.9 years [20,21,22,23]). Therefore, studies with adult JIA-patients and longitudinal studies should be conducted. 

As expected, TMJ dysfunction was more common in JIA-patients: 52.5% instead of 13% in the general population had a diagnosis according to RDC/TMD (Table 4). Especially the prevalence of group III diagnosis (arthralgia, osteoarthrosis, osteoarthritis) is clearly lower in epidemiological data (1.4%) than in JIA-patients (45.8%). Surprisingly, the most frequent diagnosis referring to RDC/TMD was osteoarthrosis (35.6%) not osteoarthritis (5.1%). Only one other study using the RDC/TMD diagnosis system is available [36], which examined only 15 JIA patients. Thereby the most common diagnosis of the TMJs was arthralgia (56.7%), 10% showed osteoarthritis and 6.7% osteoarthrosis. The RDC/TMD is a validated and standardized diagnostic protocol for temporomandibular disorders. Nevertheless, the consensus-based recommendations for the orofacial examination in JIA do not recommend the usage of any combination of clinical outcome measures in research studies as they have only moderate diagnostic value in predicting TMJ inflammation [37]. However, these recommendations were already not available when the current study was planned and conducted. Both, the RDC/TMD as well as Helkimo index are neither validated for clinical assessment in JIA patients nor adapted for children. Accordingly, the diagnoses according to the RDC/TMD have to be questioned, especially regarding the fact that JIA is not considered in this protocol. In general, for interpretation of the data, one must be aware that the RDC/TMD can only give information about TMJ dysfunction not about real TMJ involvement [38,39]. Radiographically, 47% of participants showed TMJ deformities in the current study. This rate is very similar to the TMJ involvement in other studies [40,41] that had comparable mean disease duration (about five years). Clearly longer (13.1 years) or shorter (2 years) disease duration was reported to lead to clearly higher (60%) or lower (28.9%) TMJ involvement [42,43]. These high rates combined with less anamnestic pain (29%) and the potentially fatal orthodontic consequences [4,5] underline the large importance of detailed functional examination of these patients. Furthermore, JIA patients with TMJ involvement report higher functional disability and lower oral health-related quality of life [44,45]. In particular, early diagnosis of TMJ involvement is important as these severe clinical signs can be improved by early orthodontic treatment [46,47,48,49]. Positive anamnestic or clinical findings should be followed by imaging methods, because these patients showed significantly higher radiographic scores, potentially showing an irreversible destruction of joint tissue.

The current study revealed associations between an increased CRP and higher values of gingival inflammation and radiographic deformities of the TMJ. Available data are inhomogeneous, whereby the associations of gingival and/or periodontal inflammation to CRP seemed unclear [20,21]. Thereby, it is questionable whether there would be a causative link between oral and systemic inflammation. Restrictions in oral hygiene due to pain and reduced motor skills might lead to increased oral inflammation and could also cause these associations. A positive correlation between increased CRP and radiographic deformities of the TMJ was found for rheumatoid arthritis in previous studies, and is thereby in line with literature [50,51]. In contrast, ANA-positive patients showed lower scores for gingival and periodontal inflammation in the current study. A possible explanation could be that ANA is more often positive in oligoarticular forms [52]. HLA-B27-positive patients showed higher PSI scores. This possible association between this genetic marker and periodontitis has already been shown under animal-experiment conditions [53] and in another study [54]. These surface antigens could be risk indicators for periodontitis in JIA-patients (HLA-A01, HLA-B27, HLA-B35, DRB3 (positive) and HLA-A24 (negative)) [54]. However, a causative relationship cannot be confirmed by the current study. 

The current study could not reveal correlations between other disease related parameters, for example, affected joints, limited mouth opening, and temporomandibular pain to oral health findings. Even though some trends are obvious (higher dmf-t/DMF-T in patients with limited mouth opening and in patients with temporomandibular pain (Table 3)), there was no significant difference probably because of the small number of participants in these subgroups. Other studies could show such possible associations: JIA patients with attachment loss had more joints with limited movement [20], JIA patients with more affected joints of the upper limb showed more gingival inflammation [55], JIA patients taking NSAIDs had less gingival inflammation [21]. However, data is very inconsistent, probably for the same reason. For an adequate risk assessment large multicenter studies should be conducted. 

Strengths and limitations: The current study investigated JIA-patients very extensively. Dental, periodontal and functional findings were assessed in the same patients by one calibrated examiner. Furthermore, the temporomandibular joint was investigated not only radiographically but also by a detailed clinical functional analysis referring to the RDC/TMD. For complete consideration of the whole complexity of these patients, the orthodontic examination of possible dentofacial anomalies is missing in this study. Particularly, various disease parameters as blood parameters, medication and affected joints were assessed. Thereby, possible associations could be revealed. Nevertheless, a more detailed evaluation of the current status of JIA as current disease activity and active joints would have been desirable. For interpretation of the data, it has to be considered that no contrast-enhanced magnetic resonance imaging (MRI) has been performed. This method can be seen as the gold standard, because only this imaging technique allows evaluation of active inflammation in TMJ [56]. Neither by clinical [38] nor by radiographic [57] findings TMJ arthritis can be assessed, especially in an early stage. Therefore, it has to be stated that in this study no TMJ arthritis could be diagnosed and all associations or correlations only refer to TMJ dysfunction or radiographic TMJ deformity. 

**Table 4:** Comparison of oral health parameters of JIA-patients of the current study to epidemiological data.

		JIA-Patients (Current Study)	Epidemiologic Data	
			Jordan, Micheelis 2016 (DMS V) [33]	Wu, Hirsch 2010 [58]
**Study characteristics**	Study period	2012–2014	2013–2014	2000–2001
	Sample size (*n*)	59	1468	561
	Female gender (*n* (%))	42 (71.2)	715 (48.7)	281 (50.1)
	Age in years (mean ± SD)	12.7 ± 3.1	12 ± 0	14.7 ± 1.1
**Caries** (*n* = 59) mean ± SD	dmf-t/DMF-T	2.6 ± 3.0	0.5	n/a
	d-t/D-T	0.9 ± 1.5	0.1	n/a
	m-t/M-T	0.0 ± 0.0	0.1	n/a
	f-t/F-T	1.6 ± 2.5	0.3	n/a
**Gingival inflammation (maximum value)** (*n* = 55) in %	PBI 0	1.8	22.3	n/a
	PBI 1	23.6	30.6	n/a
	PBI 2	27.3	37.9	n/a
	PBI 3	36.4	9.0	n/a
	PBI 4	10.9	0.1	n/a
**Diagnosis according to RDC/TMD** (*n* = 59) in %	No diagnosis	47.5	n/a	87.0
	Group I	5.1	n/a	0.4
	Group II	13.6	n/a	10.7
	Group III	45.8	n/a	1.4

dmf-t/DMF-T: decayed-, missing- and filled-teeth index, PBI: papilla bleeding index, PSI: periodontal screening index, SD: standard deviation, RDC/TMD: Research Diagnostic Criteria for Temporomandibular Disorders.

The most important methodological limitation of this study is the lack of an age and gender matched healthy control group. For estimating the significance of the findings, the fifth German oral health study [33] (DMS V) was used as surrogate control group. This large study (*n* = 1.468) gives representative epidemiologic data for the German general population and therefore can be seen as adequate national reference. The data are comparable because of the similar age (DMS V: 12 years, current study: mean age = 12.7 ± 3.1) and the corresponding study period (DMS V: 2013–2014, current study: 2012–2014). Furthermore, in contrast to control groups, these epidemiological data are population representative and do not have bias as prevention-oriented controls. Unfortunately, no data about the socioeconomic status of the JIA patients have been assessed. Consequently, only comparison to the average of all socioeconomic status is possible, even if the DMS V showed a significant influence of the socioeconomic status on the prevalence of caries and gingival inflammation. As the DMS V did not assess dental biofilm, periodontal screening index nor functional parameters, functional parameters were compared to another population-based study representative for the German population [58]. As only a few epidemiologic studies on temporomandibular disorders in adolescents are available, here discrepancies regarding age and study period are higher. The present gender ratio (71.2% female) is typical for JIA (63.8% female according to prevalence data [2]). With 59 patients an adequate cohort size could be reached. Anyway, the case numbers for subgroups as for example of affected joints and medication were too small for revealing possible associations. The distinct diversity of patients suffering from rheumatic diseases in general and JIA patients in particular, for example, in exact diagnosis referring to the categories of the JIA-classification, disease duration, disease activity, degree of severity, affected joints and medication, poses a big challenge in composing homogenous groups in adequate sample size for studies. Especially, revealing the effect of different medication is complex, as its change during therapy is common. These differences complicate the comparison of the different studies.

Furthermore, the validity remains reduced, because the cross-sectional study design does not allow any conclusions about causalities. Meanwhile, consensus-based recommendations for the examination of JIA in research studies are available to ensure comparable results between investigations [37]. These recommendations were not available when the current study was conducted, which is a further limitation, as these recommendations should be followed in clinical examinations. 

## 5. Conclusions

Adolescents suffering from JIA show high levels of caries experience and gingival inflammation. TMJ dysfunction is very common. Consequently, this rheumatic disease requires habitual and prompt involvement of a dentist as oral health expert, whereby special dental care programs would be recommendable. Moreover, disease related blood values could be associated to oral inflammation and TMJ deformities. Dentists should be involved for successful medical care of these patients. 

## Figures and Tables

**Table 1:** Characteristics of study participants (*n* = 59) including disease related parameters and oral findings.

Parameters			Number of Patients (*n* (%))/Years (a)
**Gender**		Male	17 (28.8)
		Female	42 (71.2)
**Age in years** (mean ± SD)			12.7 ± 3.1
**Duration of disease** (mean ± SD)			5.1 ± 4,5
**Type of JIA** ^1^		Oligoarthritis	37 (62.7)
		Polyarthritis	22 (37.3)
**Disease Related Parameters**			
**Medication** (*n* = 57)	**Medication**	**Total Number of Patients (*n* (%))**	**Current Number of Patients (*n* (%))**
	NSAIDs	49 (83)	n/a
	Glucocorticoids	6 (10)	2 (3)
	Methotrexate	27 (46)	7 (12)
	Sulfasalazine	2 (3)	1 (2)
	Adalimumab Etanercept, Indometacin	7 (12)	4 (7)
**Blood analysis**	**Blood Parameter**	**Positive in Number of Patients (*n* (%))**	**Negative in Number of Patients (*n* (%))**
	ANA ^2^ (*n* = 51)	31 (61)	20 (39)
	HLA-B27 (*n* = 41)	10 (24)	31 (76)
	RF ^3^ (*n* = 43)	1 (2)	42 (98)
	Anti-dsDNA (*n* = 33)	2 (6)	31 (94)
	CRP ^4^ (*n* = 52)	26 (50)	26 (50)
**Affected joints**^5^ (*n* = 59)	**Joints**	**Affected in Number of Patients (*n* (%))**	
	Upper extremity in total	30 (50.8)	
	Finger	21 (35.6)	
	Wrist	19 (32.3)	
	Elbow	15 (25.4)	
	Shoulder	3 (5.1)	
	Other than upper extremity	57 (96.6)	
**Oral Findings**			
**Dental findings** (*n* = 59)	**Parameters**	**Mean ± SD**	
	dmf-t/DMF-T	2.6 ± 3.0	
	d-t/D-T	0.9 ± 1.5	
	m-t/M-T	0.0 ± 0.0	
	f-t/F-T	1.6 ± 2.5	
**Gingival inflammation**^6^ (*n* = 55)	**Maximal Score**	**Number of Patients (*n* (%))**	
	PBI 0	1 (1.8)	
	PBI 1	13 (23.6)	
	PBI 2	15 (27.3)	
	PBI 3	20 (36.4)	
	PBI 4	6 (10.9)	
**Periodontal screening**^6^ (*n* = 57)	**Maximum Value**	**Number of Patients (*n* (%))**	
	PSI 0	3 (5.3)	
	PSI 1	15 (26.3)	
	PSI 2	16 (28.1)	
	PSI 3	23 (40.4)	
	PSI 4	0 (0.0)	

^1^ Classification according to the number of affected joints in the first six months of disease (oligoarthritis: ≤4, polyarthritis: >4); ^2^ ANA cut-off titer: 1:80; ^3^ current blood test RF positive; ^4^ CRP cut-off value: 8 mg/L; ^5^ a joint counted as affected if it has ever been involved during disease duration; ^6^ extended orthodontic appliances precluded measurement of gingival inflammation in four and of periodontal screening index in two cases; SD: standard deviation; NSAIDs: Nonsteroidal anti-inflammatory drugs; n/a: not applicable; positive: result of the blood parameter elevated over the normal range; negative: result of the blood parameter in normal range; ANA: Antinuclear antibodies; HLA-B27: Human leukocyte antigen B27; RF: Rheumatoid factor; Anti-dsDNA: Anti-double stranded DNA antibodies; CRP: C-reactive protein; dmf-t/DMF-T: decayed-; missing- and filled-teeth index; PBI: papilla bleeding index; PSI: periodontal screening index.

**Table 2:** Anamnestic, clinical and radiographic findings.

Parameter		Number of Patients (*n* (%))
**Anamnestic findings** (*n* = 59)	Positive in total	24 (41)
	Joint noise	14 (24)
	Reduced mouth opening capacity	9 (15)
	Pain	17 (29)
**Clinical findings** (*n* = 59)	Positive in total	32 (54)
	Joint noise	20 (34)
	Reduced mouth opening capacity	15 (25)
	Pain with muscle palpation	4 (7)
	Pain with joint palpation	10 (17)
**Diagnosis according to RDC/TMD** (*n* = 59)	No diagnosis	28 (47.5)
	Arthralgia	3 (5.1)
	Osteoarthritis	3 (5.1)
	Osteoarthrosis	21 (35.6)
	Myofascial Pain without limited opening	3 (5.1)
	Myofascial Pain with limited opening	0 (0)
	Disc displacement with reduction	3 (5.1)
	Disc displacement without reduction with limited opening	5 (8.5)
	Disc displacement without reduction without limited opening	0 (0)
**Helkimo’s Clinical Dysfunction Index** (*n* = 59)	D_i_0: Absence of clinical symptoms of dysfunction	15 (25.0)
	D_i_I: light clinical symptoms of dysfunction	27 (46.0)
	D_i_II: moderate clinical symptoms of dysfunction	8 (13.5)
	D_i_III: severe clinicalsymptoms of dysfunction	9 (15.5)
**Radiographic findings** (*n* = 51)	Score 0	27 (53)
	Score 1	15 (29)
	Score 2	5 (10)
	Score 3	4 (8)

RDC/TMD: Research Diagnostic Criteria for Temporomandibular Disorders.

**Table 3:** Association of affected joints, reduced mouth opening capacity and pain with dental findings (dmf-t/DMF-T).

Parameters			Number of Patients	Mean dmf-t/DMF-T ± SD	Mean Rank	*z*-Value	*p*-Value

**Affected joints**	Upper extremity	Positive	30	2.6 ± 2.8	30.4	–0.201	0.840
		Negative	29	2.6 ± 3.2	29.6		
	Finger	Positive	21	2.3 ± 2.5	29.5	–0.186	0.853
		Negative	38	2.7 ± 3.2	30.3		
	Wrist	Positive	19	2.3 ± 2.8	28.5	–0.472	0.637
		Negative	40	2.7 ± 3.1	30.7		
	Elbow	Positive	15	2.7 ± 2.9	31.6	–0.427	0.670
		Negative	44	2.5 ± 3.0	29.5		
	Shoulder	Positive	3	6.0 ± 4.4	46.5	–1.744	0.081
		Negative	56	2.4 ± 2.8	29.1		

**Reduced mouth opening capacity**	Anamnestic	Negative	50	2.4 ± 2.4	30.2	–0.215	0.830
		Positive	9	3.6 ± 5.2	28.9		
	Clinical	Negative	44	2.3 ± 2.2	29.7	–0.222	0.824
		Positive	15	3.5 ± 4.5	30.8		

**TMJ pain**	Anamnestic	Negative	42	2.3 ± 2.4	29.77	–0.162	0.871
		Positive	17	3.1 ± 4.1	30.56		
	Clinical	Negative	47	2.2 ± 2.4	28.71	–1.163	0.245
		Positive	12	3.9 ± 4.4	35.04		

dmf-t/DMF-T: decayed-, missing- and filled-teeth index, SD: standard deviation, TMJ: temporo-mandibular joint.
